# Potential risk factors associated with human encephalitis: application of canonical correlation analysis

**DOI:** 10.1186/1471-2288-11-120

**Published:** 2011-08-22

**Authors:** Jemila S Hamid, Christopher Meaney, Natasha S Crowcroft, Julia Granerod, Joseph Beyene

**Affiliations:** 1 Clinical Epidemiology and Biostatistics, McMaster University, Hamilton, Canada; 2 Pathology and Molecular Medicine, McMaster University, Hamilton, Canada; 3 Dalla Lana School of Public Health, University of Toronto, Toronto, Canada; 4 Family and Community Medicine, University of Toronto, Toronto, Canada; 5 Public Health Ontario, Toronto, Canada; 6 Health Protection Agency, Centre for Infections, London, UK

## Abstract

**Background:**

Infection of the CNS is considered to be the major cause of encephalitis and more than 100 different pathogens have been recognized as causative agents. Despite being identified worldwide as an important public health concern, studies on encephalitis are very few and often focus on particular types (with respect to causative agents) of encephalitis (e.g. West Nile, Japanese, etc.). Moreover, a number of other infectious and non-infectious conditions present with similar symptoms, and distinguishing encephalitis from other disguising conditions continues to a challenging task.

**Methods:**

We used canonical correlation analysis (CCA) to assess associations between set of exposure variable and set of symptom and diagnostic variables in human encephalitis. Data consists of 208 confirmed cases of encephalitis from a prospective multicenter study conducted in the United Kingdom. We used a covariance matrix based on Gini's measure of similarity and used permutation based approaches to test significance of canonical variates.

**Results:**

Results show that weak pair-wise correlation exists between the risk factor (exposure and demographic) and symptom/laboratory variables. However, the first canonical variate from CCA revealed strong multivariate correlation (ρ = 0.71, se = 0.03, p = 0.013) between the two sets. We found a moderate correlation (ρ = 0.54, se = 0.02) between the variables in the second canonical variate, however, the value is not statistically significant (p = 0.68). Our results also show that a very small amount of the variation in the symptom sets is explained by the exposure variables. This indicates that host factors, rather than environmental factors might be important towards understanding the etiology of encephalitis and facilitate early diagnosis and treatment of encephalitis patients.

**Conclusions:**

There is no standard laboratory diagnostic strategy for investigation of encephalitis and even experienced physicians are often uncertain about the cause, appropriate therapy and prognosis of encephalitis. Exploration of human encephalitis data using advanced multivariate statistical modelling approaches that can capture the inherent complexity in the data is, therefore, crucial in understanding the causes of human encephalitis. Moreover, application of multivariate exploratory techniques will generate clinically important hypotheses and offer useful insight into the number and nature of variables worthy of further consideration in a confirmatory statistical analysis.

## Background

Encephalitis is a complex clinical syndrome of the central nervous system (CNS) associated with fatal outcome or severe permanent damage including cognitive and behavioral impairment and epileptic seizures [1-5]. It is often acute, although symptoms may progress rapidly, causing severe debilitation to patients including otherwise healthy children [2,3]. Lewis and Glaser define encephalitis as an acute CNS dysfunction with radiographic or laboratory evidence of brain inflammation [2]. There is no standard laboratory diagnostic strategy for investigation of encephalitis and even experienced physicians often are uncertain about the cause, appropriate therapy and prognosis [1-3,6].

Despite being identified worldwide as an important public health concern, retrospective studies on encephalitis are very few and studies often focus on particular types (often with respect to causative agents) of encephalitis (West Nile, Japanese, etc.). However, there are relatively more studies in the pediatric population [2,3,7,8]. Moreover, current knowledge about encephalitis is limited to descriptive statistics. As a result, a comprehensive understanding of human encephalitis, as generated through high quality evidence-based studies and statistical analyses is limited and much of the current knowledge base lacks generalizability [2,9-11].

Encephalitis is characterized by fever, headache and altered level of consciousness together with seizures and focal neurological findings in some cases [1,3,11]. Using data from the same prospective study presented in this paper, our group previously identified fever, personality and behavioural change, headache and lethargy, as the main characteristics of human encephalitis [10,11]. It was also shown that diagnostic variables such as abnormal brain scan and cerebrospinal fluid measurements are also indicators of encephalitis. Seizures, focal neurological deficits, stiff neck, urinary symptoms, respiratory symptoms and gastro-intestinal symptoms have also been previously shown to be associated with encephalitis [1,2,11]. Fowler et al., in retrospective study of paediatric encephalitis, found that fever and encephalopathy were the main disease characteristics in a Swedish sample [3].

Encephalitis is a rare disease, with annual incidence ranging between 3.5-7.4 cases per 100,000 persons worldwide [1,2,12]. It affects people of all ages; however, the condition is more common in children, the elderly and persons with a weakened immune system (e.g. HIV/AIDS patients and patients undergoing cancer treatment). Encephalitis is known to affect both sexes; however, most studies have indicated a slightly higher incidence rate in males [1,13-15]. The epidemiology of encephalitis is difficult to summarize since few population based studies exist, many causal pathogens are capable of inducing encephalitis-like symptoms and most cases go unreported to health authorities. Consequently, many details about its epidemiology have yet to be explained [1,2,10].

To date, infection of the CNS is considered to be the major cause of encephalitis and more than 100 different pathogens have been recognized as causative agents [1,10]. However, an estimated 32-85% of cases have unknown disease etiology [1,16-20]. For instance, about 85% of the 189 cases in a study conducted in Minnesota, USA are of unknown cause [20]. In a California based study, about 65% of the 334 cases are of unknown etiology [18]. In a study conducted in the UK, about 60% of 700 cases are of unknown etiology [16]. Among the known causes, Herpes Simplex Virus (HSV) has been recognized as the most common etiology [1,10,20]. Viruses, bacteria, fungi as well as parasites can cause encephalitis [1-3]. Rarely, encephalitis can also be triggered by brain injury, brain tumor, drug reactions and lead poisoning. The main infectious causes of encephalitis are listed in a review paper by Granerod and Crowcroft [1].

In many parts of the world, viral infections of the central nervous system are often spread via vector-borne infection, such as mosquito bites and tick bites; however, animal-to-human interactions also can facilitate disease spread (e.g. raccoon feces, cat scratches, animal bites) and human-to-human transmission is also possible. Bacteria causing encephalitis can also spread through animal contact and water exposure. Possible risk factors associated with encephalitis and disease pathologies are provided in Lewis and Glaser [2].

A number of other infectious and non-infectious conditions present with similar symptoms and hence a challenge lies in distinguishing encephalitis from other disguising conditions [1,2,6]. Exploration of human encephalitis data using advanced multivariable statistical modelling approaches that can capture the inherent complexity in the data is, therefore, crucial for elucidating the causes of human encephalitis. Moreover, application of multivariate exploratory techniques will generate clinically important and better focused hypotheses that would benefit encephalitis researchers in reducing the number of variables to be considered for further confirmatory statistical analysis. This will ultimately lead towards better evidence-based clinical practices, including: diagnosis, prognosis discovery and development of novel therapeutic options.

In this paper, we use canonical correlation analysis (CCA) to explore the relationship between a set of exposure variables that are potential risk factors and a set of symptom and diagnostic variables in encephalitis. The symptom and diagnostic variables considered in this paper include variables that are previously identified as main indicators of encephalitis as well as those with a potential to be associated with the disease. Our data consist mostly of binary variables (presence or absence of a particular attribute) and as a result, the usual correlation matrix which is particularly designed for continuous measurements is not appropriate. We therefore propose to use a correlation matrix based on Gini's idea of variance or likeability for categorical variables.

## Methods

### Study population and data description

Data consists of 268 patients recruited from 24 hospitals/neurological centers in three geographical locations across England (South West, London, North West). Measurements from 16 symptom, 6 diagnostic (3 from cerebrospinal fluid, 2 from brain scans/images and 1 electroencephalography) and 13 exposure variables were recorded. Age, gender, duration of illness and length of hospital stay were also available. Most of the variables in the study are binary indicating presence or absence of attributes; others have been dichotomized before performing the CCA analysis. Age is dichotomized where one group consisting of young children (age ≤ 10), and another group consisting of older children and adults (> 10 years). Duration of illness is dichotomized as short (≤ 100 days) and long (> 100 days) and length of hospital stay is dichotomized as short (≤ 50) and long (> 50). These cutoff values are determined using results from analysis of univariate distributions. Variables included in our study are listed in Table 1. More details about the UK encephalitis study can be found in the original paper [10].

**Table 1:** List of the two sets of variables: One set consisting of 13 exposure and 2 demographic variables, and a second set consisting of 18 symptoms, clinical and 6 diagnostic variables

Exposure and Demographic Variables	Symptom/clinical and Diagnostic Variables
Animal contact	Lethargy
Tick bite	Personality/behavioral changes
Mosquito bite	Seizure
Insect bite	Stiff neck
Immunization	Headache
Recent infection	Irritability
Travel abroad	Fever
Travel within UK	Focal neurological findings
Raw fish	Coma
Untreated water	Neurological signs
Head trauma	Gastrointestinal symptoms
Sick person contact	Respiratory symptoms
Water Exposure	Confusion
Age	Photophobia
Gender	Rash
	Urinary symptoms
	Duration of illness
	Length of Hospital Stay
	Abnormal white blood cell count (WCC)
	Abnormal magnetic resonance imaging (MRI)
	Abnormal computed tomography (CT)
	Abnormal electroencephalography (EEG)
	Abnormal glucose
	Abnormal protein

### Methods

We used canonical correlation analysis (CCA) to investigate the relationship between the set of exposure and demographic variables (***X***) and the set of symptom, clinical and diagnostic variables (***Y***) in human encephalitis.

#### Canonical Correlation Analysis (CCA)

Consider two sets of variables ***X_p _****= {****x***_*1*_*, ****x***_*2*_*, *. . ., ***xp****} *and ***Y***_*q *_*= {****y***_*1*_*, ****y***_*2*_*, *. . ., ***y***_*q*_*}, *measured on n individuals, where p and q represent the number of variables in each set. Canonical correlation analysis seeks to determine the optimal set of *min (p, q) *linear combinations (called canonical variates), ***a'x *= **∑ ***a_i _x_i_*** and ***b'y *= **∑ ***b_j _y_j_***, from sets ***X***_*p *_and ***Y***_*q *_which produce maximum correlation [21-25]. That is, the method finds two vectors ***a = (a*_*1*_*, a*_*2 *_*,*..., *a*_*p*_) **and ***b = (b*_*1*_*, b*_*2*_*, *. . ., *b*_*q*_) **such that the following correlation is maximized.

(1) $$Corr\left(a\prime x,b\prime y\right)=\frac{aS_{xy}b}{\sqrt{a\prime S_{xx}a}\sqrt{b\prime S_{yy}b}}$$

Where, ***S***_*xx *_and ***S***_*yy *_are the within-set covariance matrices for ***X ***and ***Y***, respectively, and ***S***_*xy *_is the between set covariance matrix. The solution is obtained by solving the following two eigenvalue problems [23,24]

$$\left(S_{yy}^{-1}S_{yx}S_{xx}^{-1}S_{xy}-\lambda I\right)a=0$$

$$\left(S_{xx}^{-1}S_{xy}S_{yy}^{-1}S_{yx}-\lambda I\right)b=0,$$

where, the Eigen-values λ, which sometimes are denoted by r^2^, represent the squared canonical correlations. The set of Eigen-vectors *(**a**, **b**) *corresponding to the leading eigenvalue are solutions to equation (1). The first canonical covariate is therefore the one which explains most of the relationship. CCA has been successfully applied in medical and epidemiological research [26,27]

#### Covariance/Correlation matrix for categorical data

Since data in this study consist mostly of binary variables (presence or absence of a particular attribute), the usual correlation matrix, which is particularly designed for continuous measurements would not be an appropriate choice. Covariance or correlation matrices for categorical data have been previously considered by many and several formulations have been proposed to assess the strength of association between two categorical variables. Here we use the covariance/correlation matrix proposed by Okada et al. [29,30]. Their approach is a generalization of Gini's definition of variance or likeability for categorical data, which is also known as Gini's index [28-33].

Let X = {x_1_, x_2_, ..., x_p_} where x_i_'s are categorical variables measured on n individuals. The ij^th ^element of the variance-covariance matrix **V **(the covariance between x_i _and x_j _when i≠j and V_ii _is the variance of x_i_) is calculated as

$$V_{ij}=\max\left(Q_{ij}\left(L\right)\right),$$

where,

$$Qij\left(L\right)=\frac{1}{2n^{2}}\underset{a}{\sum }\underset{b}{\sum }\left(x_{ia}-x_{ib}\right)\prime L\left(x_{ja}-x_{jb}\right),$$

Where, **L **is an orthogonal matrix (orthogonal transformation) [30], in our case L = 1. When calculating variance, for instance, ***x_ia _*= *x_ib _*= 1 **if ***x_ia _*≠ *x_ib _***and ***x_ia_* - *x_ib_*= 0 **if *x_ia_*= *x_ib_*. The ij^th ^element of the correlation matrix **R **can then be calculated as

$$R_{ij}=\frac{V_{ij}}{\sqrt{V_{ii}}\sqrt{V_{jj}}}$$

Simplified formulas for two special cases (binary and trinomial variables), using 2 × 2 and 3 × 3 contingency tables, can be found in Okada et al. [30,33]. We implemented the above variance-covariance/correlation formula in the R statistical software and used it in our CCA analysis. Pairwise available data were used when missing values occur.

Statistical analysis is performed using the Canonical Correlation Analysis (CCA) and Significance Tests for Canonical Correlation Analysis (CCP) libraries in the R software package [34-36]. Parametric multivariate tests are not appropriate since our data consists of binary variables and hence violates the multivariate normality assumption. We, therefore, used a non-parametric permutation approach and calculated standard errors and p-values based on 10,000 permutations.

## Results and Discussion

Our data set consists of 268 patients (152 from North West England, 94 from London and 22 from South West), of which 263 met the case definition (the case definition criteria are presented in the original paper our group recently published [10]), 208 of these patients are confirmed encephalitis cases (40 of the 208 cases are meningoencephalitis patients). We focused on these 208 confirmed encephalitis patients for the CCA analysis in this paper; however, for comparison purposes, we have also performed the analysis on the 263 patients for whom the case definition was met. Summary statistics for our data on encephalitis patients is presented in Table 2.

**Table 2:** Descriptive statistics for data on the 208 confirmed encephalitis patients

Variables	Present	Absent	Missing
*Exposure Variables and Demographic*			
Sex (male)	113 (54.3%)	95 (45.7%)	0 (0%)
Age (≤ 10)	55 (26.4%)	146 (70.2%)	7 (3.4%)
Animal Contact	101 (48.6%)	95 (45.7%)	12 (5.8%)
Tick Bite	7 (3.4%)	188(90.4%)	13 (6.3%)
Mosquito Bite	13 (6.3%)	182 (87.5%)	13 (6.3%)
Insect Bite	14(6.7%)	179 (86.1%)	15 (7.2%)
Immunization	14 (6.7%)	182 (87.5%)	12 (5.8%)
Recent Infection	78 (37.5%)	113 (54.3%)	17 (8.2%)
Travel Abroad	27 (13%)	174 (83.7%)	7 (3.4%)
Travel UK	31 (14.9%)	164 (78.8%)	13 (6.3%)
Raw Fish	7 (3.4%)	184(88.5%)	17 (8.2%)
Untreated Water	6 (2.9%)	185 (88.9%)	17 (8.2%)
Water Exposure	38 (12.3%)	157 (75.5%)	13 (6.3%)
Head Trauma	23 (11.1%)	173 (83.2%)	12 (5.8%)
Sick Person Contact	54 (26%)	136(65.4%)	18 (8.7%)
*Symptom and Diagnostic Variables*			
Abnormal CT	51(24.5%)	123 (59.1%)	34 (16.3%)
Abnormal MRI	102 (49%)	69 (33.2%)	37 (17.8%)
Abnormal EEG	100 (48.1%)	20 (9.6%)	88(42.3%)
Abnormal Glucose	46(22.1%)	84 (40.4%)	78 (37.5%)
Abnormal Protein	124 (59.6%)	71 (34.1%)	13 (6.3%)
Abnormal WCC	160 (76.9%)	42 (20.2%)	6 (2.9%)
Lethargy	116 (55.8%)	92(44.2%)	0 (0%)
Irritability	77(37%)	131 (63%)	0 (0%)
PB Change	133 (63.9%)	75 (36.1%)	0 (0%)
Seizure	105(50.5%)	103(49.5%)	0 (0%)
Stiff Neck	46 (22.1%)	162(77.9%)	0 (0%)
Headache	125 (60.1%)	83(39.9%)	0 (0%)
Fever	162 (77.9%)	46(22.1%)	0 (0%)
Focal-Neurological	76 (36.5%)	132(63.5%)	0 (0%)
Coma	8 (3.8%)	200(96.2%)	0 (0%)
Neurological	63 (30.3%)	145 (69.7%)	0 (0%)
GI Symptoms	103(49.5%)	105(50.5%)	0 (0%)
Respiratory	42 (20.2%)	166 (79.8%)	0 (0%)
Confusion	74(35.6%)	134(64.4%)	0 (0%)
Rash	25 (12%)	183 (88%)	0 (0%)
Photophobia	16 (7.7%)	192 (92.3%)	0 (0%)
Urinary	21(10.1%)	187 (89.9%)	0 (0%)
Hospital Stay (≤ 50 days)	145(69.7%)	60(28.8%)	3(1.4%)
Duration of illness (≤ 100 days)	167(80.3%)	31(14.9%)	10(4.8%)

The results in Table 2 show that men are at a slightly higher (54%, n = 113) risk of encephalitis than women (46%, n = 95). This is in agreement with previous findings [13-15]. Most of the encephalitis patients are children and young adults (median age = 30, IQR = 45) where a large proportion of the patients are children of age ≤ 10 (26%, n = 55) indicating that young children are at higher risk of developing encephalitis. The age distribution is quite uniform after age 10 where approximately equal proportions of patients (9.6%, n = 20) are observed in 10 years age intervals. We, therefore, used 10 as a cutoff point when dichotomizing age for the CCA analysis.

Our results show that the majority of encephalitis patients (69.7%, n = 145) had been hospitalized for ≤ 50 days (median = 27; IQR: 43) and duration of illness is less than 100 days (median = 37, IQR = 46.25) for large proportion (80%, n = 167) of the patients. Consequently, we used 50 days and 100 days as cutoffs when dichotomizing hospital stay and duration of illness for CCA analysis, respectively.

**Figure 1:**
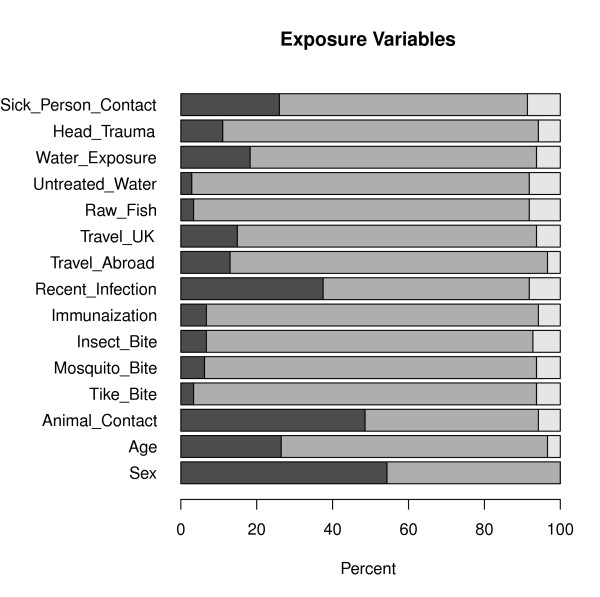
**Bar plot showing the distribution of exposure variables for the 208 encephalitis patients**. Dark grey, light grey and white represent percentage of patients who are exposed, not exposed and missing exposure status, respectively.

**Figure 2:**
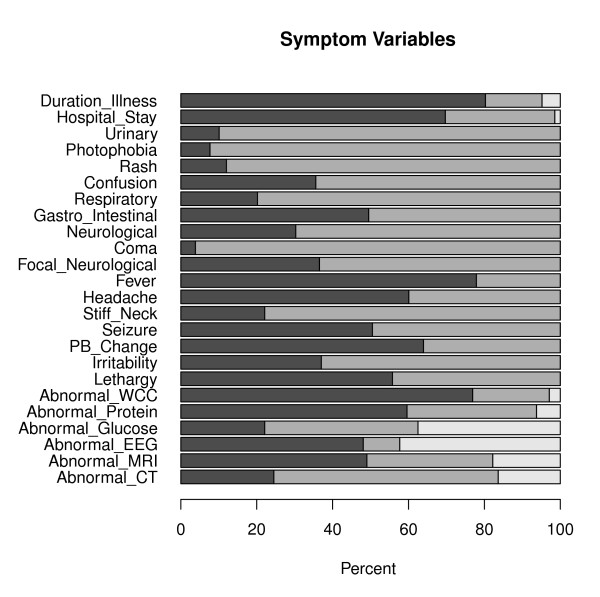
**Bar plot showing the distribution of symptom and laboratory measurements for the 208 encephalitis patients**. Dark grey, light grey and white represent percentage of patients for whom the symptoms are present, absent and symptom status missing, respectively.

Overall, data on the encephalitis patients is sparse in nature where large proportion of zeroes (absence) than ones (presence) is observed for most of the variables (Figures 1 and 2). This is particularly the case for the exposure variables (Figure 1) with the exception of animal contact (48.6% exposed), recent infection (37.5% of the patients have had recent infection) and sick person contact (26%). For instance, the percentage of patients exposed to tick and mosquito bites are only 3.4% (n = 7) and 6.3% (n = 13), respectively. A considerable percentage of patients had water exposure (18.3%) and have experienced head trauma (11.1%).

On the other hand, symptom and diagnostic variables have relatively larger event rates (Figure 2) where variables with the smallest rates are coma and photophobia which were observed on only 3.8% (n = 8) and 7.7% (n = 16) of the patients, respectively. Fever and abnormal white blood cell count (abnormal WCC)are indicated as the two main characteristics of encephalitis where 77.9% and 76.9% of the patients had fever and abnormal WCC, respectively (Figure 2, Table 2). The results also show that personality and behavioral change, headache, lethargy and abnormal protein are the next most frequently occurring characteristics of encephalitis. Some missingness are observed in the exposure variables (Figure 2); however, a significant amount of missing data are observed in diagnostic variables where measurements from EEG and Glucose were missing for 42.3% (n = 88) and 37.5% (n = 78) of the patients, respectively (Table 2 Figure 2). Consequently, abnormal EEG, although previously shown to be one of the main indicators of encephalitis, is observed on only half of the patients (48.1%). Nevertheless, among patients with available EEG measurements (n = 120), 83.3% (n = 100) of them have abnormal EEG which is in agreement with previous findings. This is mainly because the diagnostic decision tree often leads clinicians to carry out an EEG in patients with a high likelihood of it being abnormal. One of the triggers is seizures, for example. So patients with EEGs are a particular clinical cluster of their own.

**Figure 3:**
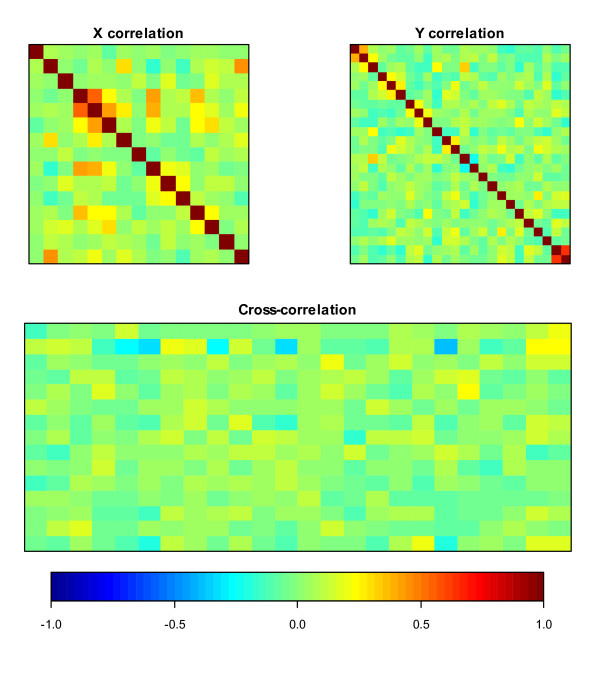
**Correlations within and between two sets of variables where **X **represents exposure and demographic variables, and **Y **consist of symptom, clinical and diagnostic variables**.

Heatmaps of within and between set correlations are presented in Figure 3 where dark blue and dark red colors indicate very strong correlations (a color indicator bar with ranges of correlations is presented under the heatmaps). Figure 3 indicates that, weak to moderate (-0.22-0.63) pair-wise correlations exist both within and between the ***X ***and ***Y ***sets of variables, in general where, the largest correlations are observed between length of hospital stay and duration of illness (0.63), and between tick and insect bites (0.55).

CCA produced min (p, q) = 15 canonical variates; *p = 15 *is the number of variables in the ***X ***set and *q = 24 *is the number of variables in the ***Y ***set. However, only the first canonical variate is statistically significant at α = 0.05 level. We will, therefore, discuss only the first canonical variate in this paper.

The cross-correlation matrix displayed in Figure 3 shows that weak pair-wise correlation exists between the risk factor (exposure and demographic) and outcome (symptom, clinical and diagnostic) variables. However, the first canonical solution/variate from CCA revealed strong multivariate correlation (ρ = 0.71, standard error (se) = 0.03, p-value = 0.013) between the two sets. We found a moderate correlation (ρ = 0.54, se = 0.02) between the variables in the second canonical variate, however, the value is not statistically significant (p-value = 0.68).

The first canonical solution consists of two sets of variables: the linear combination of ***X ***set variables (exposure and demographic features) and the linear combination of the ***Y ***set variables (symptom, clinical and diagnostic features). Individual canonical loadings (structural coefficients) between these two sets of variables with their corresponding canonical variates are presented in Table 3.

**Table 3:** Canonical loadings of individual variables in their respective canonical variates for the first canonical solution of the CCA

Canonical Loadings (Structural Coefficients)			
***Exposure and Demographic Variables***		***Symptom and Diagnostic Variables***	

Sex (male)	0.03	Abnormal CT	0.07
Age (≤ 10)	0.94	Abnormal MRI	0.08
Animal Contact	-0.04	Abnormal EEG	0.22
Tick Bite	0.05	Abnormal Glucose	-0.19
Mosquito Bite	0.11	Abnormal Protein	-0.39
Insect Bite	-0.08	Abnormal WCC	-0.52
Immunization	0.27	Lethargy	0.27
Recent Infection	-0.10	Irritability	0.28
Travel Abroad	-0.06	PB Change	-0.36
Travel UK	-0.13	Seizure	0.24
Raw Fish	-0.05	Stiff Neck	-0.12
Untreated Water	-0.05	Headache	-0.51
Water Exposure	0.18	Fever	-0.03
Head Trauma	0.13	Focal-Neurological	-0.06
Sick Person Contact	0.47	Coma	0.001
		Neurological	-0.24
		GI Symptoms	0.08
		Respiratory	0.13
		Confusion	-0.50
		Rash	0.11
		Photophobia	-0.18
		Urinary	-0.11
		Hospital Stay (≤ 50 days)	0.30
		Duration of illness (≤ 100 days)	0.30

A correlation of ρ = 0.71 (p-value = 0.01) is obtained for the first canonical correlation.

**Figure 4:**
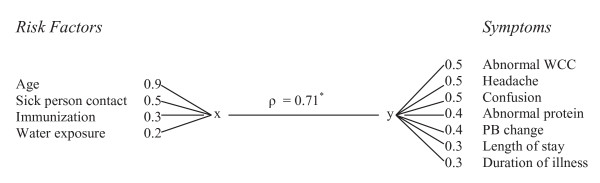
**The top ranked variables in the first canonical solution and absolute value of their canonical loadings**. A multivariate correlation of ρ = 0.71 (p-value = 0.01, indicated in the figure by *) is obtained between the two sets of variates.

The top ranked variable in the exposure set is age (loadings = 0.94) indicating that age contributed large amount of variation (88%) in the first canonical variate of exposure sets and hence the driving variable for the canonical variate The cross loading for age also shows that a considerable amount (45%) of the variation in the canonical variate of symptoms is explained by age. This result is in agreement with previous findings that showed that children are at an increased risk of developing encephalitis compared to adults. Sick person contact and immunization also contributed considerably towards the first canonical variate with ladings of 0.47 and 0.27; and cross loadings of 0.34 and 0.22, respectively. The contribution of the rest of the exposure variables towards the variation in the first canonical variate is negligible. Variables that contributed the least include animal contact and sex, where only 0.25% the variation in the first canonical variate was attributed to these variables. Variables that contribute to the first canonical variates of both sets are provided in a simple "finger plot" presented in Figure 4.

Among the symptom and diagnostic variables, abnormal WCC, headache and confusion are the three top ranked variables contributing 27%, 26%, and 25% of the variation in the first canonical variate of the symptom sets, respectively. The other variables with a considerable contribution towards the first canonical variate are abnormal protein, PB change, length of hospital stay and duration of illness, explaining 15%, 12%, 9% and 9% of the variation, respectively. The canonical cross loadings also indicate that symptom variables, provided in Figure 4, explain considerable amount of the variation in the first canonical variate of the exposure sets.

Fever, although present in the majority of the patients (77.9%, Table 2), does not contribute much towards the first canonical variates, explaining only 0.04% and 0.16% of the variation in the symptom and exposure variates, respectively.

We also performed CCA on the 263 patients who met the case definition criteria as presented in the original paper [10]. In general, the pattern observed in the within and between correlations for this data set is similar to those obtained for the 208 confirmed encephalitis cases where weak to moderate correlations exist between the variables. A correlation of ρ = 0.68 (p-value = 0.007) was obtained between sets of variables in the first canonical solution. The second canonical solution resulted in ρ = 0.54 (p-value = 0.19). Overall, the canonical loadings for ***X ***and their rankings are similar to those presented in Table 3 and Figure 4, respectively. Therefore, our analysis based on 263 patients indentified the same sets of exposure variables to be strongly associated with symptom, clinical and diagnostic variables.

Redundancy coefficients indicate that very small amount of the variation in the original symptom variables were explained by the exposure canonical variates. Only 6% of the variation in the symptom variables is explained by the first exposure canonical variate; 5% by the second canonical variate and 4% by the third. This indicates that, the variation in the symptoms might be caused by host factors rather than environmental and exposure factors. The idea that characteristics of the host may be more important than the pathogen is consistent with the observation that for some causes, such as herpes simplex virus (HSV), encephalitis is a rare outcome of a common infection. Another possible hypothesis, that might be drawn from our results, is the possibility that exposure and symptom variables might provide independent information towards understanding the etiology of encephalitis. Further case-control type of analysis based on exposure, symptom and host factors might shed light to better understanding of factors that might help facilitate diagnosis and treatment of encephalitis patients.

## Conclusion

We performed exploratory multivariate analysis using CCA to study associations between two sets of variables in encephalitis patients. One set consists of exposure and demographic variables including variables that are previously indentified in the literature as potential risk factors. The second set includes symptom, clinical and diagnostic variables where some items in the set have been shown to be important clinical characteristics of encephalitis. Although pair-wise cross correlations between the two sets of variables are weak to moderate, CCA revealed strong multivariate correlation between the two sets.

Our analysis provided a set consisting of 3 exposure/demographic variables (age, sick person contact, immunization and water exposure) to be strongly associated with 7 symptom/diagnostic variables (abnormal WCC, headache, confusion, abnormal protein, personality and behavioral change, length of stay and duration of illness) to be strongly associated.

Our analysis also revealed that a very small amount of the variation in the symptom sets is explained by the exposure variables. This indicates that host factors, rather than environmental factors might be important towards understanding the etiology of encephalitis and facilitate early diagnosis and treatment of encephalitis patients.

CCA is exploratory in nature and measures associations rather than causation. However, our analysis indentified exposure variables that might be strongly associated with encephalitis and generated important hypotheses that can be investigated further to indentify risk factors that are predictive of encephalitis. A confirmatory case-control analysis involving encephalitis and non-encephalitis patients is needed to indentify risk factors and important symptom variables that can be used to facilitate diagnosis. CCA results may, however, provide insight into potentially smaller sets of variables worth investigating further. Furthermore, it is important to highlight that exposure variables such as tick bite do not occur frequently in the UK and also do not often lead to encephalitis, and so are difficult to study using conventional methods such as logistic regression analysis. CCA can, therefore, be a useful tool in indentifying risk factors associated with human encephalitis and other rare and complex diseases where regression approaches may not be optimal.

## Competing interests

The authors declare that they have no competing interests.

## Authors' contributions

JSH contributed to the design of study and methods, performed statistical analysis and interpretation of data, and wrote the manuscript. CM contributed to methods and analysis of data and participated in drafting the manuscript. NSC and JG contributed to acquisition of data and helped with critical revision of the manuscript for important intellectual content. JB contributed to the design study and methods, and participated in drafting the manuscript. All authors have read and approved the final version of the manuscript.

## Pre-publication history

The pre-publication history for this paper can be accessed here:

http://www.biomedcentral.com/1471-2288/11/120/prepub

## References

[B1] Granerod J Crowcroft N The epidemiology of acute encephalitis Neuropsychological Rehabilitation 2007 17 4 406 428 10.1080/09602010600989620 17676528

[B2] Lewis P Glaser CA Encephalitis Pediatric Reviews 2005 26 353 363 10.1542/pir.26-10-353 16199589

[B3] Flower A Stodberg T Eriksson M Wickstrom R Childhood encephalitis in Sweden: Etiology, clinical presentation and outcome European Journal of Pediatric Neurology 2008 12 484 490 10.1016/j.ejpn.2007.12.009 18313340

[B4] Misra UK Kalita J Seizures in encephalitis: Predictors and outcome Seizure 2009 18 583 587 10.1016/j.seizure.2009.06.003 19581112

[B5] Aygun AD Kabakus N Celik I Turgut M Yoldas T Gok U Guler R Long-term neurological outcome of acute encephalitis Journal of Tropical Pediatrics 2001 47 4 243 247 10.1093/tropej/47.4.243 11523767

[B6] Resznicek JE Bloch KC Diagnostic Testing for Encephalitis, Part I Clinical Microbiology 2010 32 3 17:23

[B7] Kolski H Ford-Jones EL Richardson S Petric M Nelson S Jamieson F Blaser S Gold R Otsubo H Heurter H MacGregor D Eitology of Acute Childhood Encephalitis at the hospital for sick children, Toronto, 1994-1995 Clinical Infectious Diseases 1998 26 2 398 409 10.1086/516301 9502462

[B8] Koskiniemi M Korppi M Mustonen K Rantala H Muttilainen Herrgård E Ukkonen P Vaheri A Study Group Epidemiology of encephalitis in children A prospective multicenter study 1997 156 7 541 545 10.1007/s004310050658 9243237

[B9] Starza-Smith A Talbot E Grant C Encephalitis in Children: A clinical neuropsychology perspective Neurological Rehabilitation 2007 17 4 506 527 10.1080/09602010701202154 17676532

[B10] Granerod J Ambrose HE Davies NWS Clewley JP Walsh A Morgan D Cunningham R Zuckerman M Mutton K Solomon T Ward K Lunn MPT Irani SR Vincent A Brown DWG Crowcroft NS on behalf of the UK HPA Aetiology of Encephalitis Study Group Causes of encephalitis and differences in their clinical presentations in England: a multicenter, population-based prospective study Lancet Infectious Diseases 2010 10 12 835 844 10.1016/S1473-3099(10)70222-X 20952256

[B11] Hamid JS Meaney C Crowcroft NS Granerod J Beyene J Cluster analysis for indentifying sub-groups and selecting potential discriminatory variables in human encephalitis BMC Infectious Diseases 10 364 10.1186/1471-2334-10-364 PMC3022837 21192831

[B12] Johnson RT Acute encephalitis Clinical Infectious Diseases 1996 23 219 226 10.1093/clinids/23.2.219 8842253

[B13] Cizman M Jazbec J Aetiology of acute encephalitis in childhood in Slovenia Pediatric Infectious Diseases Journal 1993 12 903 908 10.1097/00006454-199311000-00002 8265278

[B14] Lee T Tsai C Yuan C Wei C Tsao W Lee R Cheih S Huang I Chen K Encephalitis in Taiwan: A prospective hospital-based study Japanese Journal of Infectious Diseases 2003 56 193 199 14695429

[B15] Studahl M Bergstrom T Hagberg L Acute viral encephalitis in adults: A prospective study Scandinavian Journal of Infectious Diseases 1998 30 215 220 10.1080/00365549850160828 9790126

[B16] Davison KL Crowcroft NS Ramsay ME Brown DWG Andrews NJ Viral encephalitis in England, 1989-1998: What did we miss? Emerging Infectious Diseases 2003 9 234 240 12603996 10.3201/eid0902.020218 PMC2901942

[B17] Koskiniemi M Rantalaiho T Piiparinen H von Bonsdorff CH Fakkila M Jarvinen A Koskiniemi S Kinnunen E Mannonen L Muttilainen M Linnavuory K Porras J Puolakkainen M Raiha K Salonen E Ukkonen P Vaheri A Valtonen V The Study Group Infections of the central nervous system of suspected viral origin: a collaborative study from Finland Journal of NeuroVirology 2001 7 400 408 10.1080/135502801753170255 11582512

[B18] Glaser CA Gilliam S Schnurr D Forghani B Honarmand S Khetsuriani N Fischer N Cossen CK Anderson LJ In search of encephalitis etiologies-diagnostic challenges in the California encephalitis project, 1998-2000 Clinical Infectious Diseases 2003 36 6 731 742 10.1086/367841 12627357

[B19] Nicolosi A Hauser WA Beghi E Kurland LT Epidemiology of central nervous system infections in Olmsted County, Minnesota, 1950-1981 Journal of Infectious Diseases 1986 154 399 408 10.1093/infdis/154.3.399 3734490

[B20] Cinque P Cleator GM Weber T Monteyne P Sindic CJ van Loon AM The role of laboratory investigation in the diagnosis and management of patients with suspected herpes simplex encephalitis: A consensus report Journal of Neurology, Neurosurgery and Psychiatry 1996 61 339 345 10.1136/jnnp.61.4.339 PMC486570 8890768

[B21] Hotelling H Relations between 2 sets of variants Biometrika 1936 28 321 327

[B22] Mardia K Kent J Bibby J Multivariate Analysis 1979 Academic Press, San Francisco, California

[B23] Cooley W Lohnes P Multivariate Data Analysis 1971 Wiley-Interscience, Hoboken, New Jersey

[B24] McGarigal K Cushman S Stafford S Multivariate Statistics for Wildlife and Ecology Research 2000 Springer-Verlag, New York, New York

[B25] Darlington R Weinberg S Walberg H Canonical variate analysis and related techniques Review of Educational Research 1973 43 433 446

[B26] Razavi A Gill H Stal O Sundquist M Thorstenson S Ahlfeldt N Exploring cancer register data to find risk factors for recurrence of breast cancer-application of Canonical Correlation Analysis BMC Medical Informatics and Decision Making 2005 5 29 36 10.1186/1472-6947-5-29 16111503 PMC1208892

[B27] Ridderstolpe L Gill H Borga M Rutberg H Ahlfeldt H Canonical Correlation Analysis of risk factors and clinical outcomes in cardiac surgery Journal of Medical Systems 2005 29 4 357 377 10.1007/s10916-005-5895-9 16178334

[B28] Gini CW Variability and Mutability, contribution to the study of statistical distributions and relations Studi Economico-Giuridici della R. Universita de Cagliari 1912

[B29] Light RJ Margolin BH An Analysis of Variance for Categorical Data J American Statistical Association 1971 66 534 544 1971 (Review of Gini (1912) paper) 10.2307/2283520

[B30] Okada T Leung KS, Chan LW, Meng H A note on covariances for categorical data Intelligent Data Engineering and Automated Learning-IDEAL 2000 LNCS 1983 2000 150 157

[B31] Niitsuma H Okada T Covariance and PCA for Categorical Variables Lecture Notes in Computer Science 2005 3518 523 528 10.1007/11430919_61

[B32] Okada T Sum of Squares Decomposition for Categorical Data Kwansei Gakuin Studies in Computer Science 1999 14 1 6

[B33] Okada T Attribute Selection in Chemical Graph Mining Using Correlations among Linear Fragments Department of Informatics, Kwansei Gakuin University, 2-1 Gakuen, Sanda-shi, Hyogo, Japan 2008

[B34] González I Déjean S Martin PGP Baccini A CCA: An R Package to Extend Canonical Correlation Analysis Journal of Statistical Software 2008 23 12

[B35] Menzel U CCP: Significance Tests for Canonical Correlation Analysis (CCA), R Package 2009

[B36] R Development Core Team R: A language and environment for statistical computing R Foundation for Statistical Computing, Vienna, Austria 2009 http://www.R-project.org ISBN 3-900051-07-0

